# REV-ERBα regulates *Fgf21* expression in the liver via hepatic nuclear factor 6

**DOI:** 10.1242/bio.021519

**Published:** 2016-11-14

**Authors:** Rohit Chavan, Nadia Preitner, Takashi Okabe, Laureen Mansencal Strittmatter, Cheng Xu, Jürgen A. Ripperger, Nelly Pitteloud, Urs Albrecht

**Affiliations:** 1 Department of Biology, Unit of Biochemistry, University of Fribourg, Fribourg CH1700, Switzerland; 2 Service of Endocrinology, Diabetology and Metabolism, Lausanne University Hospital, Lausanne CH1011, Switzerland

**Keywords:** Circadian clock, Transcription, Physiology

## Abstract

The circadian clock contributes to the timing of many body functions including metabolism and reproduction. The hepatokine fibroblast growth factor 21 (FGF21) is a critical metabolic regulator involved in modulation of fertility. Here we show that lack of the clock component REV-ERBα elevates FGF21 levels in liver and plasma. At the molecular level, REV-ERBα modulates the expression of FGF21 via the liver-specific hepatic nuclear factor 6 (HNF6). We conclude that REV-ERBα regulates metabolism and reproduction, at least in part, via regulation of *Fgf21*.

## Abstract (precis)

**Summary:** The hepatokine Fgf21 is transcriptionally regulated by the nuclear receptor REV-ERBα with the hepatocyte-specific factor HNF6 to regulate metabolism and fertility.

## INTRODUCTION

The circadian clock allows organisms to predict daily recurring events such as sunrise, emergence of a particular food source and presence of predators. This enables them to optimally time physiological processes to the environment in order to enhance survival. For the survival of the species, successful reproduction is essential and factors enhancing the chance of reproductive success are conserved. Since the circadian system not only synchronizes physiology and behavior in an organism, but also is able to adapt to environmental changes such as seasons, it is not surprising that de-synchronization of the circadian system or mutation in clock genes can affect reproductive capacity (Ikegami and Yoshimura, 2012; Boden et al., 2013). Lack of the clock gene *Bmal1* in mice leads to irregular estrous cycles (Ratajczak et al., 2009) and impaired ovulation (Boden et al., 2010) for which *Bmal1* in ovarian theca cells appears to be important (Mereness et al., 2015). Furthermore, mice with a mutation in the *Clock* gene also display an irregular estrous cycle (Kennaway et al., 2004; Miller et al., 2004), and *Per1/Per2* mutants develop irregular estrous cycles as they age (Pilorz and Steinlechner, 2008). The nuclear receptor and clock component REV-ERBα is expressed with a circadian rhythm and represses *Bmal1* (Preitner et al., 2002), encoding a positive regulator of clock output genes. REV-ERBα also represses other genes to regulate metabolism in a tissue-dependent manner (Cho et al., 2012; Bugge et al., 2012). Hence, REV-ERBα is central to regulate complex interactions between the circadian clock and metabolism. Female mice lacking *Rev-erbα* display reduced fertility while males appear to mate and reproduce normally (Chomez et al., 2000).

Recent studies indicate that the reproductive axis and metabolism are sensitive to fibroblast growth factor 21 (FGF21) (Owen et al., 2013; Nies et al., 2016). FGF21 is a member of the endocrine FGF subfamily that is a critical metabolic regulator (Itoh, 2010; Reitman, 2007; Beenken and Mohammadi, 2009) and over-expression of *Fgf21* renders female mice infertile (Owen et al., 2013; Inagaki et al., 2007). Because the *Fgf21* promoter contains nuclear receptor response elements and E-boxes (Estall et al., 2009; Tong et al., 2010), we investigated whether *Fgf21* is regulated directly or indirectly by clock components. We find that *Fgf21* is regulated indirectly by the nuclear receptors REV-ERBα via HNF6, and the clock protein PER2 modulates the repressive function of REV-ERBα and/or the transcriptional efficiency of PPARα-mediated expression of *Fgf21*. Thus, FGF21 may be an intermediary between the clock components, metabolism and reproductive fitness.

## RESULTS

### *Rev-erbα* knock-out mice display reduced fertility

**Fig. 1.**
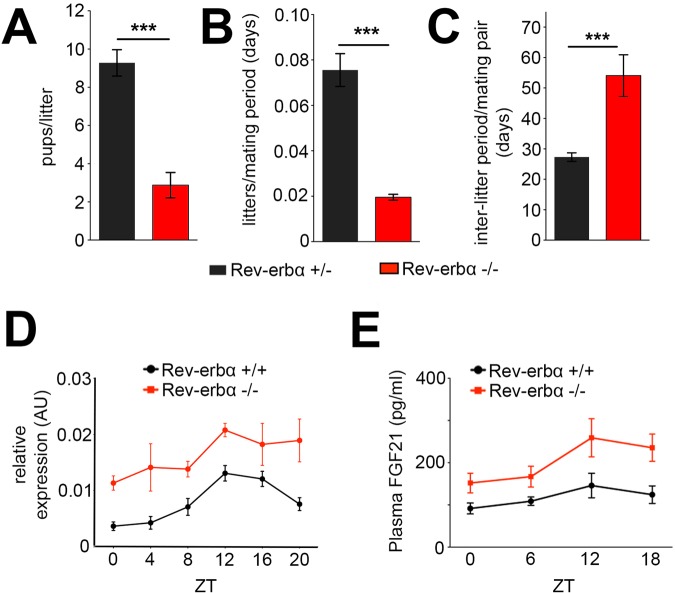
**Female *Rev-erbα^−/−^* mice display signs of reduced fertility.** (A) Rev-erbα^−/−^ (red bar) mating pairs produce significantly less pups per litter compared to Rev-erbα^+/−^ (black bar) pairs. Unpaired two-tailed *t*-test, ***P<0.0001, *n*=18 for Rev-erbα^+/−^ and *n*=8 for Rev-erbα^−/−^, *F*-test reveals no difference in variance, *F*=2.41, DFn=17, Dfd=7. (B) Number of litters per mating period is reduced in Rev-erbα^−/−^ (red bar) mating pairs compared to Rev-erbα^+/−^ (black bar) pairs. Unpaired two-tailed *t*-test, ***P<0.0001, *n*=13 for Rev-erbα^+/−^ and *n*=8 for Rev-erbα^−/−^, *F*-test reveals difference in variance, *F*=51.38, DFn=12, Dfd=7. (C) Inter-litter period is significantly longer in Rev-erbα^−/−^ (red bar) compared to Rev-erbα^+/−^ (black bar) mating pairs. Unpaired two-tailed *t*-test, ***P<0.0001, *n*=16 for Rev-erbα^+/−^ and *n*=6 for Rev-erbα^−/−^, *F*-test reveals difference in variance, *F*=8.793, DFn=5, Dfd=15. (D) Hepatic *Fgf21* mRNA in the liver is increased in Rev-erbα^−/−^ (red line) compared to Rev-erbα^+/+^ mice (black line). Two-way ANOVA with Bonferroni post-test. The two curves are significantly different, *P*=0.0008, *n*=4. (E) Plasma FGF21 protein levels are significantly increased in Rev-erbα^−/−^ (red line) compared to Rev-erbα^+/+^ animals (black line). Two-way ANOVA with Bonferroni post-test. The two curves are significantly different, *P*=0.0003, *n*=4-6. All values are mean±s.e.m.

Breeding of *Rev-erbα*^−/−^ animals in our facility revealed reduced fertility of these animals, with the average number of pups per mating pair reduced in *Rev-erbα*^−/−^ compared to the heterozygous *Rev-erbα*^+/−^ breeding pairs (Fig. 1A). Interestingly, also the number of litters per mating period was reduced (Fig. 1B) indicating that *Rev-erbα*^−/−^ breeding pairs took longer to produce offspring (Fig. 1C). This observation is consistent with a previous report describing reduced reproduction in *Rev-erbα* knock-out mice (Chomez et al., 2000).

Recent studies indicate that the reproductive axis is sensitive to fibroblast growth factor 21 (FGF21) (Owen et al., 2013). The phenotype displayed by *Rev-erbα*^−/−^ mice is reminiscent of animals overexpressing *Fgf21* (Owen et al., 2013), therefore, we tested whether *Fgf21* expression is altered in *Rev-erbα*^−/−^ mice. We found that *Fgf21* mRNA was diurnally expressed in the liver of wild-type mice in a similar fashion as *Bmal1* mRNA, a REV-ERBα target gene (Preitner et al., 2002) and in an inverted diurnal pattern compared to the E-box-driven *Per1* gene (Preitner et al., 2002) (Fig. 1D). *Fgf21* mRNA expression was increased in *Rev-erbα*^−/−^ mice at all time points over the day compared to controls (Fig. 1D). Similarly, FGF21 protein levels were also increased in plasma of *Rev-erbα*^−/−^ mice with pronounced elevation at Zeitgeber time (ZT)12 and ZT18 (Fig. 1E), but plasma FGF21 levels were not diurnal in wild-type animals (Fig. 1E). Since REV-ERBα is a nuclear receptor with repressive potential, we tested whether REV-ERBα can directly regulate the *Fgf21* promoter.

### *Fgf21* is regulated by REV-ERBα/HNF6 and PPARα/RXRα

**Fig. 2.**
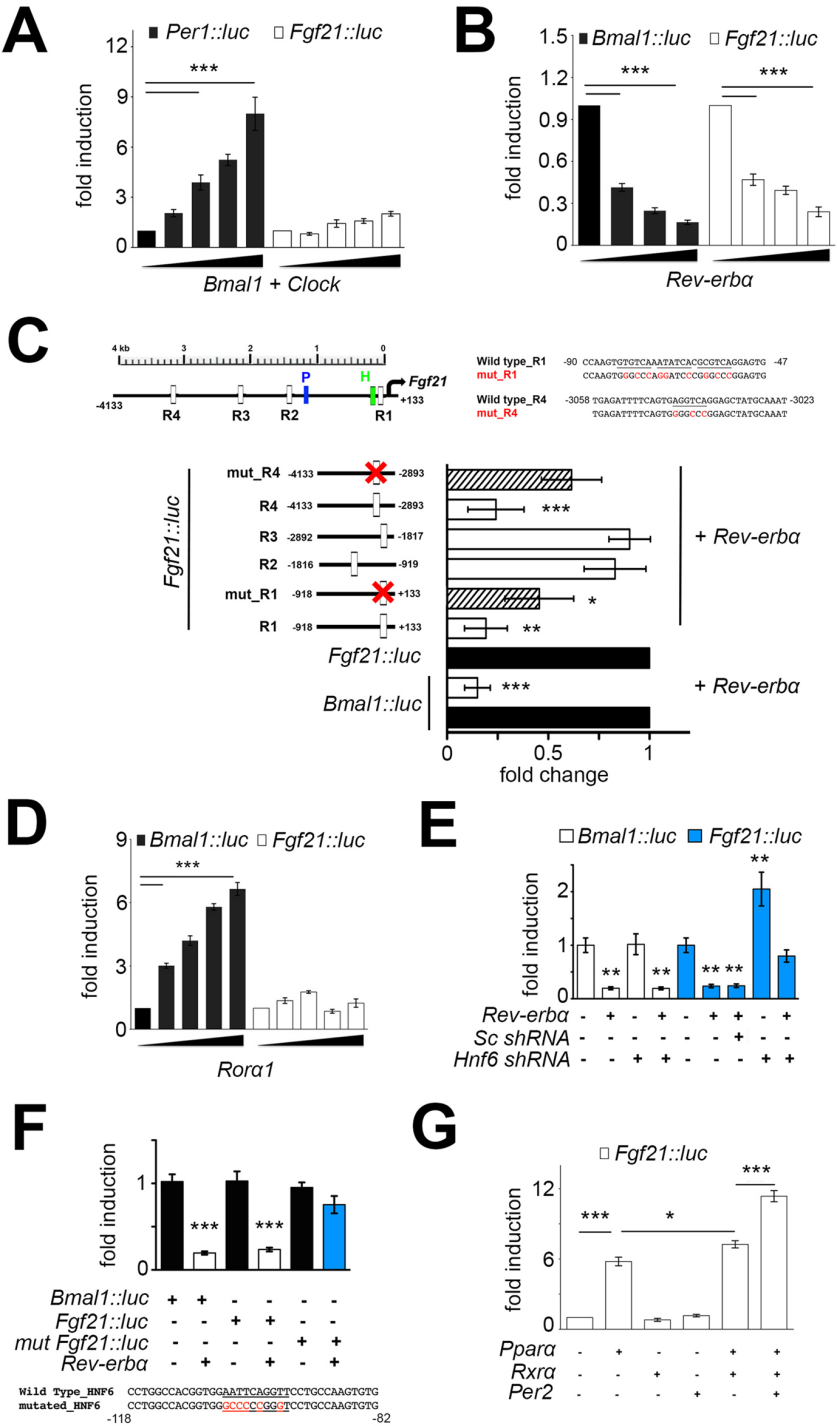
**Regulation of the *Fgf21* promoter by clock components.** (A) Dose-dependent activation of the *Per1::luc* promoter by BMAL1 and CLOCK (black bars) and no BMAL1/CLOCK activation of the *Fgf21::luc* reporter (white bars) in NIH3T3 cells (*n*=3). (B) Dose dependent repression by REV-ERBα on the *Bmal1::luc* reporter (black bars) and the *Fgf21::luc* reporter (white bars) in NIH3T3 cells (*n*=4). (C) Mutation analysis of the *Fgf21* promoter. Top left: schematic diagram showing the positions of the four ROREs (R1-R4), the PPAR element (blue) and the HNF6 binding site (green). Top right: diagram of the mutations in R1 and R4, respectively. Bottom: panels of fold change of the various constructs. Black bars: reference value for the *Bmal1::luc* and *Fgf21::luc* reporters, respectively. White bars: relative repression by Rev-erbα. Hatched bars: reduced repression by Rev-erbα on the mutated *Fgf21::luc* reporter (*n*=4). (D) Dose-dependent activating potential of RORα on the *Bmal1::luc* reporter (black bars) but not on the *Fgf21::luc* reporter (white bars) in NIH3T3 cells (*n*=3). (E) Repression of the *Bmal1::luc* (white bars) and *Fgf21::luc* (blue bars) reporters in Hepa-1c1c7 cells involves *Hnf6*. Sc shRNA, scrambled shRNA; *Hnf6* shRNA, knockdown of *Hnf6* (*n*=3). (F) Repressive potential of REV-ERBα in Hepa-1c1c7 cells on the *Bmal1::luc*, the *Fgf21::luc* and the Hnf6 site mutated *Fgf21::luc* (*mut Fgf21::luc*) reporters (*n*=3). (G) Fold induction of the *Fgf21::luc* reporter by Pparα, Rxrα and Per2 in NIH3T3 cells (*n*=4). All panels: one-way ANOVA with Bonferroni post-test. *P<0.05, **P<0.01, ***P<0.001, values are means±s.d.

Bioinformatic analysis of the *Fgf21* promoter revealed the presence of E-box elements to which the circadian clock factors BMAL1/CLOCK bind as heterodimers. Furthermore, we identified REV-ERBα binding sites, so-called putative retinoid orphan receptor elements (ROREs) and PPARα binding sites (PPARE). In a first step we tested activation of the *Fgf21* promoter by BMAL1 and CLOCK using a transactivation assay. A 3.1 kB-long fragment of the *Fgf21* promoter was linked to a luciferase reporter gene (*Fgf21::luc)* and transfected into NIH 3T3 cells along with increasing amounts of *Bmal1* and *Clock* expression vectors (Fig. 2A). As expected, BMAL1/CLOCK induced the *Per1::luc* control reporter in a dose-dependent manner. In contrast, the *Fgf21::luc* reporter was not induced, indicating that BMAL1 and CLOCK are not regulating the *Fgf21* promoter and hence are probably not directly responsible for the diurnal expression of *Fgf21* mRNA observed in Fig. 1D. Since BMAL1 and CLOCK activate not only *Rev-erbα* but also *Pparα* (Canaple et al., 2006), the expected repression of the *Fgf21::luc* reporter is probably compensated by the activating potential of PPARα (Inagaki et al., 2007).

We tested the repressive potential of REV-ERBα on the 3.1 kB *Fgf21* promoter, containing four ROREs and one PPARE, designated as R1-R4 and P in Fig. 2C, respectively. REV-ERBα repressed the *Bmal1::luc* reporter as expected, and also repressed the *Fgf21::luc* reporter (Fig. 2B) in a dose-dependent manner. Mutation of the R1 element in the *Fgf21::luc* reporter reduced the repressive potential of REV-ERBα (Fig. 2C), which is consistent with previous findings that REV-ERBα may repress *Fgf21* expression via this RORE site (Estall et al., 2009). Interestingly, however, the repression was not completely reversed by the mutation of R1, suggesting that additional promoter elements are most likely involved in the REV-ERBα-mediated repression of *Fgf21*. Therefore, we tested the R2 and R3 elements as potential REV-ERBα binding sites, but no repression through these two sites was observed (Fig. 2C). In contrast, the R4 element appeared to be involved in the repression of *Fgf21* by REV-ERBα, as mutation of this element partially abolished the repressive potential of REV-ERBα (Fig. 2C). Hence, the ROREs R1 and R4 may be the sites of REV-ERBα binding at the *Fgf21* promoter, regulating its expression. Since the classical mechanism of REV-ERBα-mediated repression involves competition with the transcriptional activator RORα, we tested whether RORα activates the *Fgf21::luc* construct. As expected, RORα could activate the *Bmal1::luc* reporter in a dose-dependent manner (Fig. 2D), however, the *Fgf21::luc* reporter did not or only poorly respond to RORα (Fig. 2D). Therefore, we suspected that *Fgf21* is indirectly regulated by REV-ERBα via hepatic nuclear factor 6 (HNF6), a second mechanism through which REV-ERBα can act (Zhang et al., 2015) (see also the presence of an HNF6 binding site H in Fig. 2C). Transactivation experiments in Hepa-1c1c7 cells revealed that REV-ERBα suppresses the *Bmal1::luc* reporter in an HNF6 independent manner, because *Hnf6* shRNA did not affect this repression (Fig. 2E); although *Hnf6* mRNA and protein was strongly repressed with *Rev-erbα* expression unaffected (Fig. S1). Interestingly, *Hnf6* shRNA induced *Fgf21* mRNA expression, indicating that HNF6 is mediating a repressing activity on the *Fgf21* promoter (Fig. S1). Therefore, we tested the influence of HNF6 on the REV-ERBα repressive function of the *Fgf21* promoter observed in Fig. 2B. We found that *Hnf6* shRNA, but not scrambled shRNA, increased the luciferase activity in the *Fgf21::luc* reporter, suggesting that HNF6 is a direct modulator of this promoter, and the lack of it increases baseline expression of *Fgf21*. Accordingly, the repression by REV-ERBα on this promoter only partially repressed activation and in absence of HNF6, REV-ERBα alone did not repress the *Fgf21::luc* reporter below baseline levels (Fig. 2E). This suggests that repression of the *Fgf21* promoter is most likely the result of cooperation between HNF6 and REV-ERBα. Interestingly, there is an HNF6 binding site (Zhang et al., 2015) close to the RORE designated as R1 in Fig. 2C. Mutating this HNF6 binding site abolished REV-ERBα mediated repression of the *Fgf21::luc* reporter (Fig. 2F), indicating that repression of this reporter by REV-ERBα requires the binding of HNF6.

Because we identified a PPAR binding site (P in Fig. 2C) between the RORE R1 and R2, we tested whether PPARα, together with its heterodimerizing partner RXRα, may modulate the *Fgf21::luc* reporter. We observed that PPARα activated this reporter and combined with RXRα this induction was even greater (Fig. 2G). Addition of Per2 increased this activation further (Fig. 2G), indicating that PPARα, RXRα and Per2 have an activating function on the *Fgf21* promoter.

### Binding of REV-ERBα, HNF6 and PPARα to the *Fgf21* promoter in liver

**Fig. 3.**
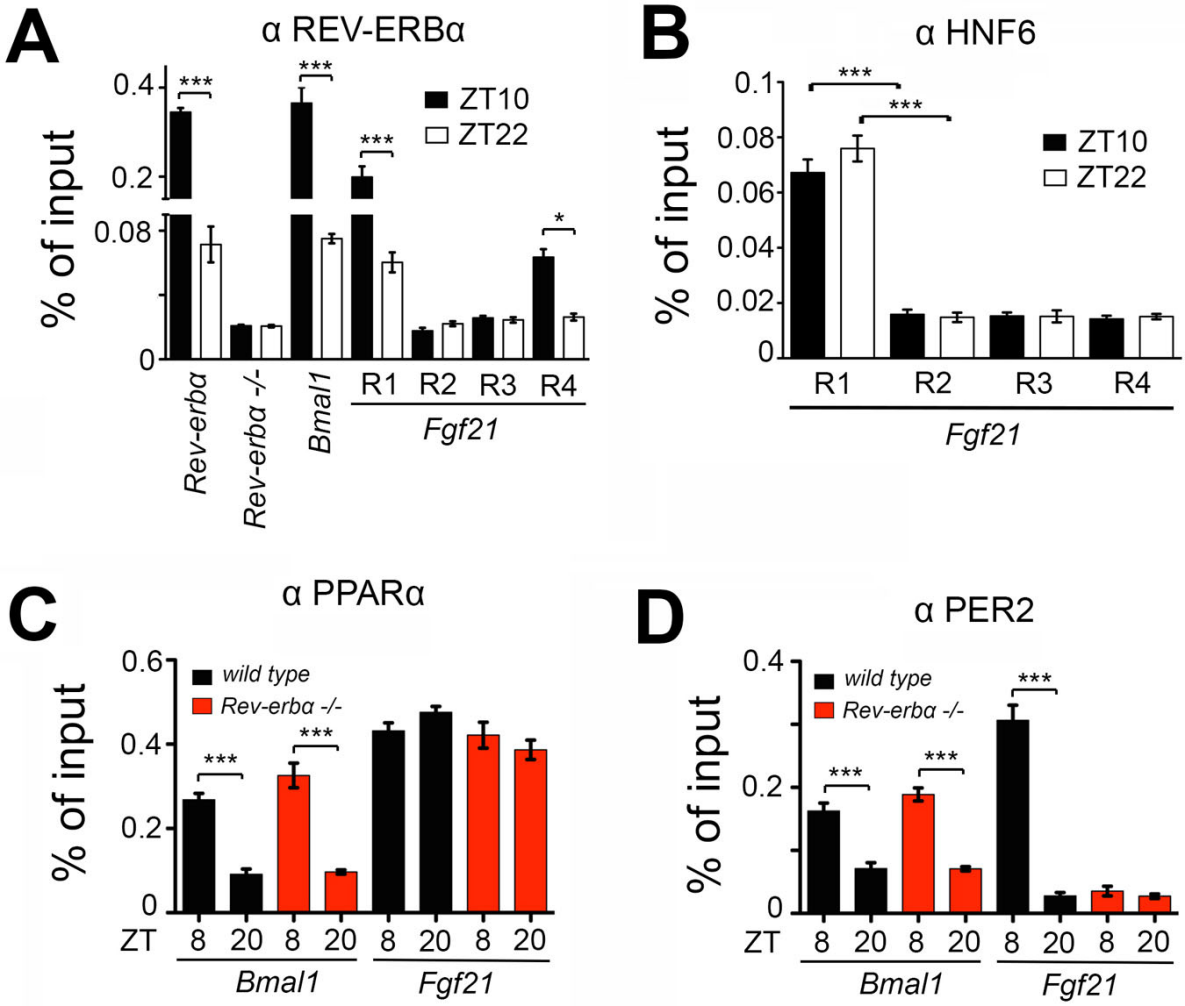
**Chromatin immunoprecipitation of REV-ERBα, HNF6, PPARα and PER2 on the *Fgf21* promoter of liver chromatin.** (A) Binding of REV-ERBα to its own promoter and to the *Bmal1* and *Fgf21* promoters at Zeitgeber time (ZT) 10 (black bars) and ZT22 (white bars), respectively. (B) Binding of HNF6 to the R1-R4 elements on the *Fgf21* promoter at ZT10 and ZT22. (C) Binding of PPARα on the *Bmal1* and *Fgf21* promoters in the liver of wild-type (black bars) and Rev-erbα^−/−^ (red bars) animals at ZT8 and ZT20. (D) Binding of PER2 on the *Bmal1* and *Fgf21* promoters in the liver of wild-type (black bars) and Rev-erbα^−/−^ (red bars) animals at ZT8 and ZT20. All panels: two-way ANOVA with Bonferroni post-test, *n*=3, *P<0.05, ***P<0.001; values are means±s.e.m.

In order to test whether the regulation of the *Fgf21* promoter by REV-ERBα, HNF6 and PPARα can occur in liver tissue, we performed chromatin immunoprecipitation (ChIP) experiments. Chromatin from livers of female mice was isolated and antibodies against REV-ERBα, HNF6 and PPARα were used to identify the promoter sequences in *Fgf21* bound by these transcription factors. We tested the four RORE-containing regions (R1-R4) in the *Fgf21* promoter for binding of REV-ERBα (Fig. 3A). We observed the strongest binding to the R1 element (which included also the HNF6 site) with weaker binding to the R4 element and no binding to the R2 and R3 elements. Interestingly, the binding to R1 and R4 was time-of-day dependent with more REV-ERBα binding at ZT10 than ZT22 (Fig. 3A), which is consistent with the expression pattern of *Fgf21* (Fig. 1D,E). The pattern of REV-ERBα binding was similar compared to the *Bmal1* and *Rev-erbα* promoter controls (Fig. 3A). In contrast, binding of HNF6 to the *Fgf21* promoter was not time-of-day dependent and only occurred in the R1 element, which includes the HNF6 binding site. R2, R3 and R4 did not show HNF6 binding (Fig. 3B). Similar to HNF6, PPARα binding to the *Fgf21* promoter was not time-of-day dependent, with strong binding at both ZT8 and ZT20 in both wild-type and *Rev-erbα^−/−^* mice (Fig. 3C). Interestingly, PPARα binding to the *Bmal1* promoter was time-of-day dependent (Fig. 3C), indicating that the mechanism of regulation by PPARα of the *Fgf21* promoter is different from the regulation of the *Bmal1* promoter.

Because PER2 can bind to both REV-ERBα and PPARα (Schmutz et al., 2010), we tested PER2 binding to the *Fgf21* promoter. We observed that PER2 binds in a time-of-day dependent manner to both the *Bmal1* and *Fgf21* promoters in wild-type mice, however, only to the *Bmal1* promoter in *Rev-erbα*^−/−^ animals (Fig. 3C). This indicates that binding of PER2 to the *Fgf21* promoter depends on the presence of REV-ERBα and/or PPARα.

## DISCUSSION

There is strong evidence that the circadian clock influences fertility and therefore reproductive fitness in mice (Miller and Takahashi, 2013; Sellix and Menaker, 2010). We observed that *Rev-erbα^−/−^* mice produce less pups and have less litters associated with a longer inter-litter period (Fig. 1A-C); however, the mechanisms linking the clock with reproduction are not well understood. We provide evidence that FGF21, which regulates metabolism (reviewed in Nies et al., 2016) and modulates fertility (Owen et al., 2013), may be one of the links.

Animals overexpressing FGF21 show reduced fertility, which is similar to the phenotype of *Rev-erbα^−/−^* animals. This similarity is further highlighted by our finding that expression of *Fgf21* mRNA, as well as FGF21 protein, is elevated in *Rev-erbα^−/−^* mice (Fig. 1D,E). Hence, *Rev-erbα^−/−^* animals can be considered as *Fgf21* overexpressors, although they overexpress *Fgf21* to a much lesser extent compared to the transgenic mice described by Owen et al. (2013).

The diurnal cycling of *Fgf21* mRNA in wild-type liver (Fig. 1D) is similar to the diurnal expression of *Bmal1*, indicating that BMAL1/CLOCK are most likely not responsible for *Fgf21* cycling, despite a previous report that described BMAL1/CLOCK-mediated activation of a 2 kb long *Fgf21::luc* reporter (Tong et al., 2010). Our own experiments indicate that our 3.1 kb *Fgf21::luc* reporter is not activated by BMAL1/CLOCK (Fig. 2A). Interestingly, ChIP-sequencing experiments revealed BMAL1 binding to the *Fgf21* promoter, however no evidence of binding for CLOCK or NPAS2 was found (Koike et al., 2012). This indicates that the *Fgf21* promoter is most likely not activated by BMAL1/CLOCK or BMAL1/NPAS2 heterodimers *in vivo*. What the role of BMAL1 binding to the *Fgf21* promoter is and whether it has activating potential with an unknown heterodimerization partner remains to be determined.

Because *Fgf21* expression in liver is increased in *Rev-erbα^−/−^* animals (Fig. 1D), reminiscent of the increased expression of *Bmal1* (Preitner et al., 2002), we tested whether the *Fgf21* promoter could be repressed by REV-ERBα. We found that *Fgf21* was repressed by REV-ERBα in a dose dependent manner, comparable to the *Bmal1* promoter (Fig. 2B). This is in line with previous findings that suggested repression of the *Fgf21* promoter by REV-ERBα (Estall et al., 2009). Interestingly, the reported RORE in that work (R1 in Fig. 2) is not the only RORE involved in the regulation of the *Fgf21* promoter as revealed by our mutation studies (Fig. 2C). It appears that at least another RORE (R4 in Fig. 2) is involved in REV-ERBα-mediated repression of the *Fgf21* promoter.

If REV-ERBα regulates *Fgf21* via direct binding to the RORE it would compete with RORα for this binding site. Therefore, we tested whether RORα induces the *Fgf21::luc* reporter in a dose-dependent manner as it does for the *Bmal1::luc* reporter. We found no dose-dependent action of RORα on the *Fgf21* promoter, in contrast to previous reports that suggested an involvement of RORα in *Fgf21* regulation (Wang et al., 2010). Of note is that no dose response curve for RORα was established in that study, which may lead to a misinterpretation of data. A recent study identified nobiletin as an agonist of ROR nuclear receptors. Application of nobiletin increased expression of ROR target genes in the liver involved in metabolism, with *Fgf21* being unaffected (He et al., 2016). This supports our observation that RORα is not involved in the regulation of *Fgf21* expression in the liver. From our data we conclude that REV-ERBα may act on the *Fgf21* promoter via another mechanism, different from the competition mechanism between REV-ERBα and RORα.

A recent study identified a second mode of action for REV-ERBα in the liver. Whereas the direct competition mechanism between REV-ERBα and ROR transcription factors provides a universal mechanism for self-sustained control of the molecular clock across all tissues, REV-ERBα uses lineage-determining factors to convey a tissue-specific rhythm that regulates metabolism tailored to the specific need of that tissue (Zhang et al., 2015). In the liver, the tissue-specific factor is HNF6 through which REV-ERBα can modulate gene expression in an HNF6-dependent fashion. In accordance with this hypothesis, we found that REV-ERBα regulates *Fgf21* expression involving HNF6 (Figs 2E,F, 3B and Fig. S1). This concept is also consistent with the observation that REV-ERBα can regulate FGF21 signaling in an adipose tissue-specific manner by directly regulating βKlotho, an essential co-receptor for FGF21 signaling (Jager et al., 2016).

In addition to HNF6 binding sites (Fig. 3B), we found PPAR elements in the *Fgf21* promoter sequence (Figs 2G and 3C). We could confirm that PPARα regulates expression of *Fgf21* (Figs 2G and 3C) as described previously (Oishi et al., 2008). Furthermore, we observed that PER2 can increase induction of the *Fgf21::luc* reporter (Fig. 2G). This increase may be partially mediated by binding of PER2 to PPARα, but lack of REV-ERBα abolishes binding of PER2 to the *Fgf21* promoter of liver chromatin (Fig. 3D), suggesting a complex regulation of the *Fgf21* promoter by PPARα and REV-ERBα with PER2 modulating the transcriptional potential of both of these nuclear receptors. This is consistent with previous observations reporting that PER2 is binding to both PPARα and REV-ERBα (Schmutz et al., 2010). Hence, PER2 may mediate the formation of a time-of-day dependent super complex containing PPARα and REV-ERBα, most likely along with additional co-factors.

**Fig. 4.**
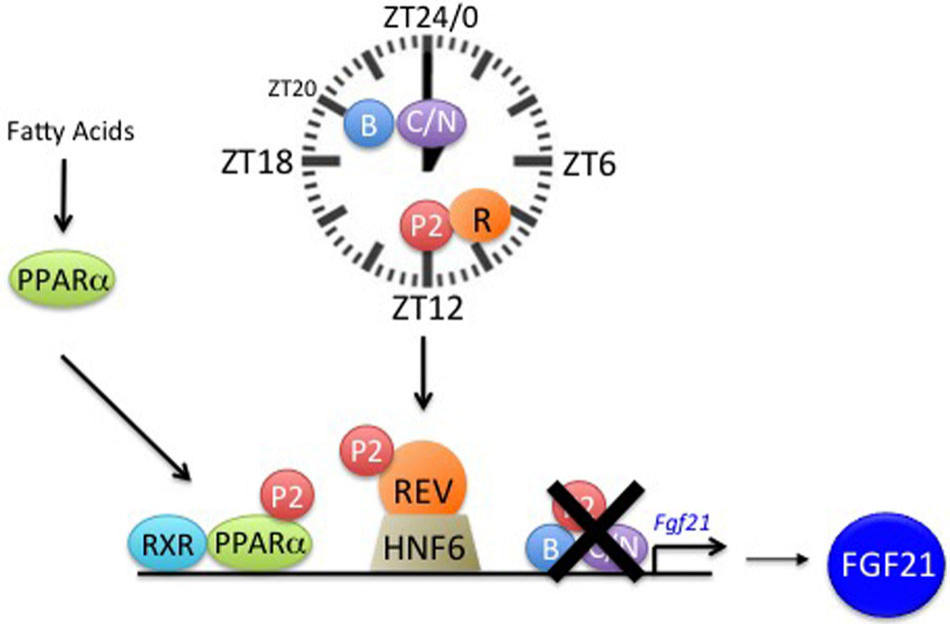
**Schematic representing clock contribution to *Fgf21* regulation in the liver.** REV-ERBα (R orange circle) represses the *Fgf21* promoter involving HNF6 (olive trapezoid). Furthermore, PPARα (green oval) with its heterodimerizing partner RXR (light blue oval) activates the *Fgf21* promoter. PER2 (P2, red circle) most likely modulates one or both of these regulations due to its capacity to bind to PPARα and REV-ERBα. Our data indicate that BMAL1 (B blue circle) and CLOCK (C/N purple oval) are not significantly involved in the regulation of *Fgf21*.

Taken together, we present evidence that REV-ERBα regulates *Fgf21* expression in the liver involving HNF6. This mechanism may be influenced by PER2 and PPARα (Fig. 4). Since FGF21 is released from the liver into the bloodstream to reach the brain influencing fertility via the hypothalamus, REV-ERBα may modulate fertility via this pathway. However, the contribution of REV-ERBα to reproductive fitness is most likely not limited to *Fgf21* regulation, but may also include additional processes, such as the regulation of ovarian biology and metabolic pathways known to be regulated by REV-ERBα (Bugge et al., 2012; Cho et al., 2012), thereby affecting fertility in an indirect manner.

## MATERIALS AND METHODS

### Animals

Animal care and handling were performed in accordance with the guidelines of the Schweizer Tierschutzgesetz (TSchG, SR455) and the Declaration of Helsinki. The protocol was approved by the state veterinarian of the Canton of Fribourg. *Rev-Erbα*^−/−^ mice were obtained from Dr U. Schibler (University of Geneva, Switzerland) and are on a mixed background 129Sv/C57BL6 (Preitner et al., 2002). Animals were maintained on 12 h light:12 h dark cycle with food and water *ad libitum*.

### Cell culture and luciferase assay

NIH3T3 and Hepa-1c1c7 mouse cells were used for *in vitro* experiments. Cells were maintained in Dulbecco's Modified Eagle Medium (DMEM), high glucose (4.5 g/l) (6429, Sigma, USA) containing 10% fetal calf serum (FCS) and 100 U/ml penicillin/streptomycin at 37°C in a humidified atmosphere containing 5% CO_2_.

Expression plasmids pSCT1-PPARα, pSCT1-RXRα, pSCT1-PER2, pSCT1-LacZ (β-galactosidase), pSCT1-REV-ERBα, pSCT1-RORα and Bmal1 luciferase construct (with PPAR regulatory site) are described (Schmutz et al., 2010).

Mouse *Fgf21* promoter is harboring four putative retinoid orphan receptor elements (ROREs) and one PPAR response element (PPRE). Five different sizes of mouse *Fgf21* promoter fragments were amplified by PCR and cloned into pGL3 basic vector (Promega, USA) using following primers: CGGTACCCTGAAGCCCCAGGTTC (R1_sense primer, KpnI site), GCTCGAGCCAAGGCAGCTGGAATTG (R1_anti-sense primer, XhoI site), CGGTACCCAGGAGGATGGAGAAC (R2_sense primer, KpnI site), GCTCGAGGAACCTGGGGCTTCAG (R2_anti-sense primer, XhoI site), CACGCGTGTCCGGCTTAGTGAAC (R3_sense primer, MluI site), GCTCGAGGTTCTCCATCCTCCTG (R3_anti-sense primer, XhoI site), CACGCGTCTCCTGTCCATTGCCAG (R4_sense primer, MluI site), GCTCGAGGTTCACTAAGCCGGAC (R4_anti-sense primer, XhoI site), CACGCGTCAGATTAAGCCACCGAGTC (sense primer, MluI site), GCTCGAGCTGGTGAACGCAGAAATAC (anti-sense primer, XhoI site). Generated luciferase reporter plasmids were designated as R1 (+133 to −918 bp), R2 (−919 to −1816 bp), R3 (−1817 to −2892 bp), R4 (−2893 to −4133 bp) and Fgf21: luc (+2 to −3099). The potential RORE and HNF6 binding elements were mutated by site-directed mutagenesis using the following primers: –90 CCAAGTGGGCCCAGGATCCCGGGCCCGGAGTG –47 (mut_R1), –3058 TGAGATTTTCAGTGGGGCCCGGAGCTATGCAAAT –3023 (mut_R4) and –117 CCTGGCCACGGTGGGCCCCCGGGTCCTGCCAAGTGTG –81 (mut_Hnf6). Luciferase assays were performed in NIH3T3 cells as described (Langmesser et al., 2008). An empty pGL3 vector was used as negative control. *Bmal1::luc* and *Per1::luc* reporter were used as positive controls.

### Knockdown of HNF6 and western blot analysis

24 h after seeding cells, HNF6-shRNA plasmid (HNF-6 sc-37937-SH, Santa Cruz Biotechnology, USA) was used for knocking-down of HNF6, according to the manufacturer's instructions. Scrambled shRNA plasmid (sc-108060, Santa Cruz Biotechnology) was used as a negative control. Knockdown efficiency was assessed 48 h post-transfection by western blotting as well as by real time PCR. Protein of transfected Hepa-1c1c7 cells was extracted using RIPA buffer (50 mM Tris-HCl pH 7.4, 150 mM NaCl, 1 mM EDTA, 0.1% SDS, 1% Triton X-100, 0.5% sodium deoxycholate containing protease and phosphatase inhibitors). Protein samples were subjected to electrophoresis on 10% SDS-polyacrylamide gels and transferred to a nitrocellulose (Amersham Protran Supported 0.45 NC, GE Healthcare). After blocking with 0.5% dry milk in PBS-Tween 0.1%, the membranes were probed with anti-HNF6 (1:500, Santa Cruz Biotechnology, sc-376167), and HSP90 (1:1000, Santa Cruz Biotechnology, sc-13119) antibodies overnight at 4°C. Anti-rabbit and -mouse HRP conjugated antibody was used as a secondary antibody. Detection of the immune complexes was performed using WesternBright Quantum system (Advansta, K-12042, USA) and quantification was done with the Quantity One analysis software (Bio-Rad).

### RNA extraction and qPCR

Total RNA was extracted from frozen liver using RNA-Bee (AMS Biotechnology, CS-105B, UK). RNA samples were treated with DNase I (Roche), and purified by phenol:chloroform extraction and ethanol precipitation. cDNA synthesis was carried out with SuperScript II (Invitrogen) and SYBR green based real-time PCR was performed for mRNA quantification using KAPA SYBR FAST (KAPA Biosystems, KK-4601, UK) and RotorGene 6000 (Corbett Research, RG-6000, Germany). All RNA samples were normalized to *Gapdh.* Primers are:

*Gapdh*:

sense: 5′-CATGGCCTTCCGTGTTCCTA-3′

antisense: 5′-CCTGCTTCACCACCTTCT TGA-3′;

*Fgf21*:

sense: 5′-ACTGCTGCTGGACGGTTA-3′

antisense: 5′-GCATCCTGGTTTGGGGAGTCCTT-3′;

Rev-erbα:

sense: 5′-CAAGGCAACACCAAGAATGTT-3′

antisense: 5′-TTCCCAGATCTCCTGCACAGT-3′;

Hnf6:

sense: 5′-CCCTGGAGCAAACTCAAGTC-3′

antisense: 5′-GGTCTCTTTCCG TGCTGCTA-3′.

The values were calculated using the double delta Ct method. 81 cycles of 10 s at 55°C with increasing increments of 0.5°C per cycle was performed for melting curve analysis. A negative control for each primer pair was included on each plate. Melting curve analysis was performed to confirm that only one product was amplified and there were not any products in negative controls. LinRegPCR was used to calculate PCR efficiencies for each sample. The RNA source, Average Ct, working annealing temperature, the average amplification efficiency and coefficients of variation are given for each gene in Table S1.

### Chromatin immunoprecipitation

For the chromatin immunoprecipitation (ChIP) experiments, mice were sacrificed at ZT10 and ZT22. The livers of mice were homogenized in 1× phosphate-buffered saline supplemented with 1% formaldehyde, incubated for 5 min at room temperature, and nuclei and chromatin prepared according to (Schmutz et al., 2010). Briefly, pure liver nuclei form each mouse were obtained by centrifugation through 2.05 M sucrose cushions and the chromatin in 500 μl of 1% SDS, 20 mM Tris pH 7.4, 150 mM NaCl, and 2 mM EDTA was fragmented by sonication (10×10 s pulses at 50% intensity using a Branson SLPe device equipped with a microtip). After a 10-fold dilution with 1.1% Triton X-100, 20 mM Tris pH 7.4, 150 mM NaCl, and 2 mM EDTA, 200 μl of chromatin were used per reaction. DNA fragments precipitated with anti-REV-ERBα antibody (1:50 dilution; SAB2101632; Sigma-Aldrich), with anti-HNF6 (G-10) (1:30 dilution; sc-376167, Santa Cruz Biotechnology), with anti-PPARα (1:25 dilution; 101710; Cayman Chemical Company, USA) or with anti-PER2 (1:25 dilution; No. 611138; BD Transduction Laboratories) were detected with the reverse transcription PCR primers and probes enlisted in Table S2. Along the samples, 1% of the input was processed and the % of input calculated as precipitated material/(amount of input ×100).
